# Combination of 3-Dimensional Virtual Reality and Hands-On Aromatherapy in Improving Institutionalized Older Adults’ Psychological Health: Quasi-Experimental Study

**DOI:** 10.2196/17096

**Published:** 2020-07-23

**Authors:** Vivian Ya-Wen Cheng, Chiu-Mieh Huang, Jung-Yu Liao, Hsiao-Pei Hsu, Shih-Wen Wang, Su-Fei Huang, Jong-Long Guo

**Affiliations:** 1 PureAroma Healing Academy Taipei Taiwan; 2 Institute of Clinical Nursing, School of Nursing National Yang-Ming University Taipei Taiwan; 3 Institute of Population Health sciences National Health Research Institutes Miaoli Taiwan; 4 Department of Health Promotion and Health Education, College of Education National Taiwan Normal University Taipei Taiwan; 5 Department of Senior Citizen Service Mackay Junior College of Medicine Taipei Taiwan

**Keywords:** three-dimensional, virtual reality, aromatherapy, older adult, happiness, stress, sleep quality, meditation, life satisfaction

## Abstract

**Background:**

In Taiwan, which has one of the most rapidly aging populations in the world, it is becoming increasingly critical to promote successful aging strategies that are effective, easily usable, and acceptable to institutionalized older adults. Although many practitioners and professionals have explored aromatherapy and identified its psychological benefits, the effectiveness of combining 3-dimensional (3D) virtual reality and hands-on aromatherapy remains unknown.

**Objective:**

A quasi-experimental trial was designed to evaluate the effectiveness of this combination in lowering perceived stress and promoting happiness, sleep quality, meditation experience, and life satisfaction among institutionalized older adults in Taiwan.

**Methods:**

A total of 60 institutionalized elderly participants either received the combined intervention or were in a control group. Weekly 2-hour sessions were implemented over 9 weeks. The outcome variables were happiness, perceived stress, sleep quality, meditation experience, and life satisfaction, which were assessed at baseline and after the intervention.

**Results:**

Generalized estimating equation (GEE) analyses indicated that the experimental group showed significant post-intervention improvements in terms of scores for happiness, perceived stress, sleep quality, meditation experience, and life satisfaction (n=48; all *P*<.001). Another GEE analysis showed that the significant improvements in the 5 outcome variables persisted in participants aged 80 years and older (n=35; all *P*<.001).

**Conclusions:**

This is the first trial to explore the effectiveness of a combination of 3D virtual reality and hands-on aromatherapy in improving older adults’ psychological health. The results are promising for the promotion of psychological health in institutionalized older adults.

**Trial Registration:**

ClinicalTrials.gov NCT04324216; https://clinicaltrials.gov/ct2/show/NCT04324216.

## Introduction

Scientific and technological advancements and the resulting improvements in human living environments and medical treatments have resulted in a gradual aging of the human population. In 2016, the global average life expectancy at birth was 72.0 years. It is estimated that by 2050, the proportion of adults over 60 will double [1,2]. Taiwan’s elderly population is one of the fastest growing in the world, and with a 14.1% elderly population rate in 2018, the country has become an aged society. This figure is expected to exceed 20% by 2026, with Taiwan then becoming a super-aged society. In order to actively plan and prepare for successful aging under these circumstances, evidence-based health promotion programs for elderly persons have gained top priority so that elderly persons can have healthy and productive lives.

However, previous studies showed poor psychological health among older residents of Taiwanese nursing homes. For example, the prevalence rates of unhappiness, poor sleep, depression, and anxiety among surveyed residents of Taiwanese nursing homes were 50% [3], 46.4% [4], 37% [5], and 26.3% [6], respectively. In addition, diminished life satisfaction and higher depression [7] were identified in nursing home residents compared with their community-dwelling counterparts, which indicates the need for psychological health interventions for the institutionalized population.

Aromatherapy, also known as essential oil therapy, is a complementary treatment that uses ingredients from different parts of plants, such as leaves, flowers, and seeds, to yield aromatic essential oils using different extraction techniques. Aromatherapy is widely used clinically in the treatment of chronic pain, anxiety, depression, cognitive disorders, insomnia, and stress-related diseases [8-10]. Studies have shown that essential oils can alleviate participants’ depression [11,12] and perceived stress [11,12] and improve sleep quality [13], meditation experience [14], and quality of life [15].

A descriptive exploratory study investigating the prevalence and type of complementary and alternative medicine (CAM) use among older Taiwanese patients with depression found that 69.6% of participants reported using at least one form of CAM, and 20.9% used aromatherapy in the past 12 months; in addition, 6.8% and 7.3% reported using aromatherapy for treating their depression weekly and daily, respectively [16]. The researchers concluded that the popularity of CAM appears to be a consequence of individuals’ preferences for a more holistic approach to health care.

Another study in Taiwan indicated that among 3 alternative remedies, aroma massage was a more effective intervention than cognitive stimulation therapy and reminiscence therapy in alleviating the agitated behavior and depressive symptoms of residents with dementia in 10 nursing homes [17]. Aroma massage has the advantage of being an easy-to-learn intervention for staff working with persons with dementia. The findings of this study contributed to clinical practice in nursing homes [17].

Three-dimensional (3D) virtual reality (VR) involves participants using devices, such as helmets and joysticks, to observe a virtual scene. This approach allows situational teaching; it provides an interactive learning environment that is not limited by time and space, thereby increasing convenience in learning and allowing real-time practice. The 3D virtual world provides rich interactions to maintain users’ attention in environments similar to the real world. Furthermore, 3D VR is interactive, integrated, and imaginative, and can be used to aid learning [18]. A strong relationship was found between depth of interaction and engagement duration, with user engagement time increasing due to the high interaction with the environment [19]. The advantages of 3D VR educational activities include avoiding the laborious traffic of learners, increasing social participation and interpersonal communication, and not being limited by time. Since these activities can be experienced and practiced any time, they are convenient for older adults with mental disabilities. Furthermore, the effectiveness of 3D VR programs for the psychological health improvement of older adults is well supported by prior literature [20].

The combination of 3D VR and hands-on aromatherapy allows for a powerful learning experience and facilitates the construction of a 3D space for aromatherapy products. There is some evidence that VR can help increase learners’ interest and motivation and effectively support knowledge transfer, since the learning process can be settled within an experiential framework [21]. In this regard, 3D VR could provide experiences with new technologies through actual use—learning in VR environments requires interaction, thus encouraging active participation rather than passivity.

Until now, it has been difficult to provide elderly persons with an opportunity to practice before performing hands-on aromatherapy activities. However, the emergence of 3D VR can solve this problem. If 3D VR can be successfully used, the difficulties elderly participants experience when engaging in hands-on aromatherapy activities might be overcome. Another advantage of a 3D VR educational activity is that it can prevent the waste of materials in hands-on activities for institutionalized older adults.

Because older adults’ hearing and hand-eye coordination are relatively poor compared with young and middle-aged adults, they are more likely to struggle with the hands-on aromatherapy activities, based on our practical experience. Thus, older adults need more workers and material support for successful engagement in hands-on activities. If the elements of 3D VR technology can be integrated into the aromatherapy intervention and reduce the burden of human and material resources, it will significantly contribute to the CAM literature. It is reasonable to conduct research that explores 2 effective strategies combined to improve the psychological health of institutionalized older adults. Therefore, the purpose of the present study was to explore the effectiveness of a combined program of 3D VR and hands-on aromatherapy in improving institutionalized older adults’ psychological health.

## Methods

### Participants

We used a quasi-experimental design, which was found to be a common study design in aromatherapy studies in a meta-analysis [22]. The research team visited 2 nursing homes and gave an introduction to the purpose and methods of the study. The nursing home which provided the consent earlier than the other was assigned as the experimental group. The other nursing home became the control group and received a compact intervention program after completion of the study. The experimental nursing home included 432 beds, and residents had a mean age of 85.2 years (male: 134/432, 31.0%). The control nursing home included 248 beds, and residents had a mean age of 84.6 years (male: 82/248, 33.1%).

A total of 30 participants were recruited from each nursing home through posters and advertisements, and the total number of participants was 60. According to Kirk [23], for an estimated effect size (estimated population mean group difference divided by the estimated population standard deviation) of .80, the approximate sample size required is 26 for each group when α is set at .05 and the power is set at .80. A previous aromatherapy study yielded significant pre-post intervention improvements in psychological health with an identical sample size [24]. Therefore, the sample size in this study (N=60) was large enough to detect intervention effects. At the end of the intervention, 6 older adults withdrew from the intervention because of serious sickness, hospitalization, etc.

The elderly participants in this study were all older than 65 years. The selection criteria included having the ability to understand verbal instructions, provide simple responses, and operate a joystick freely with at least one hand. The exclusion criteria were (1) a history of severe psychiatric conditions, (2) dementia, (3) significant visual or hearing impairment, (4) marked upper motor difficulties that could affect the participant’s ability to participate in the study, and (5) currently suffering from severe illnesses (eg, stroke, Parkinson disease).

### Ethics Statement

The study received approval from the Research Ethics Review Committee of National Taiwan Normal University (201903HM012). We confirm that we have obtained verbal permission to use images of the individuals included in this article.

### Recruitment and Baseline Procedure

**Figure 1:**
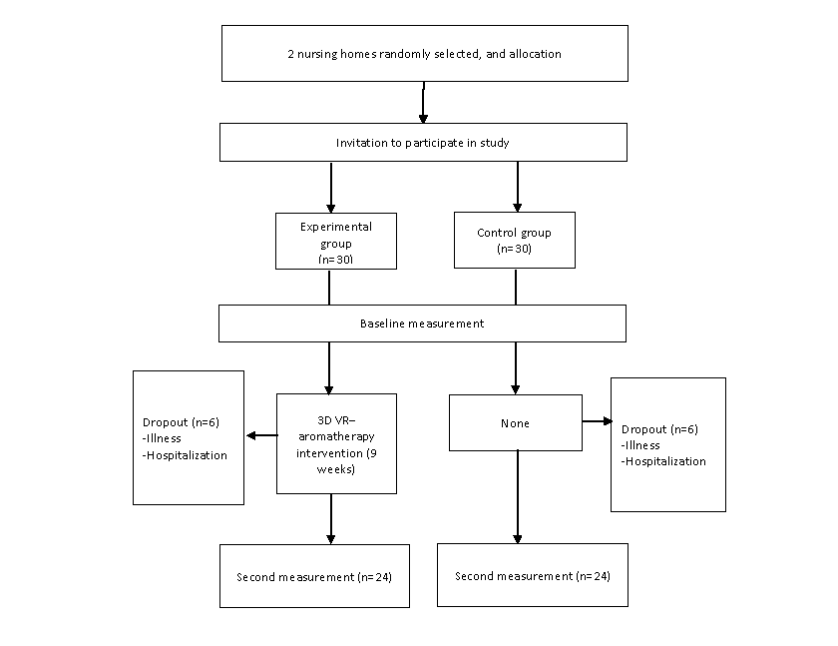
Flowchart of participant enrollment and assessment. 3D: 3-dimensional. VR: virtual reality.

A flowchart outlining participant enrollment and assessments is provided in Figure 1. After selecting the elderly care institutions, the research team approached the executive director and staff to explain the research purpose, method, and protocol. After obtaining permission to conduct the study, we posted recruitment messages to invite potential participants who met the inclusion criteria to participate in this study, and we scheduled one-on-one visits. Before obtaining written consent, we provided face-to-face explanations to potential participants, and participants then signed the consent form.

After potential participants were identified, we provided an orientation session with 3D VR and hands-on aromatherapy to test the feasibility and acceptance of the combination program. Participants indicated their appreciation of this arrangement and reported that the 3D VR program could help them perform better in the subsequent hands-on aromatherapy activities. The research team members collected their baseline data in a quiet room provided by the nursing home.

During the implementation period of the intervention, a medical professional, staff of the nursing home, and an aromatherapy professional were available to ensure the safety of the participants. The counterparts in the control group did not receive the aromatherapy intervention at the same time.

### Combination of 3D VR and Hands-On Aromatherapy

**Figure 2:**
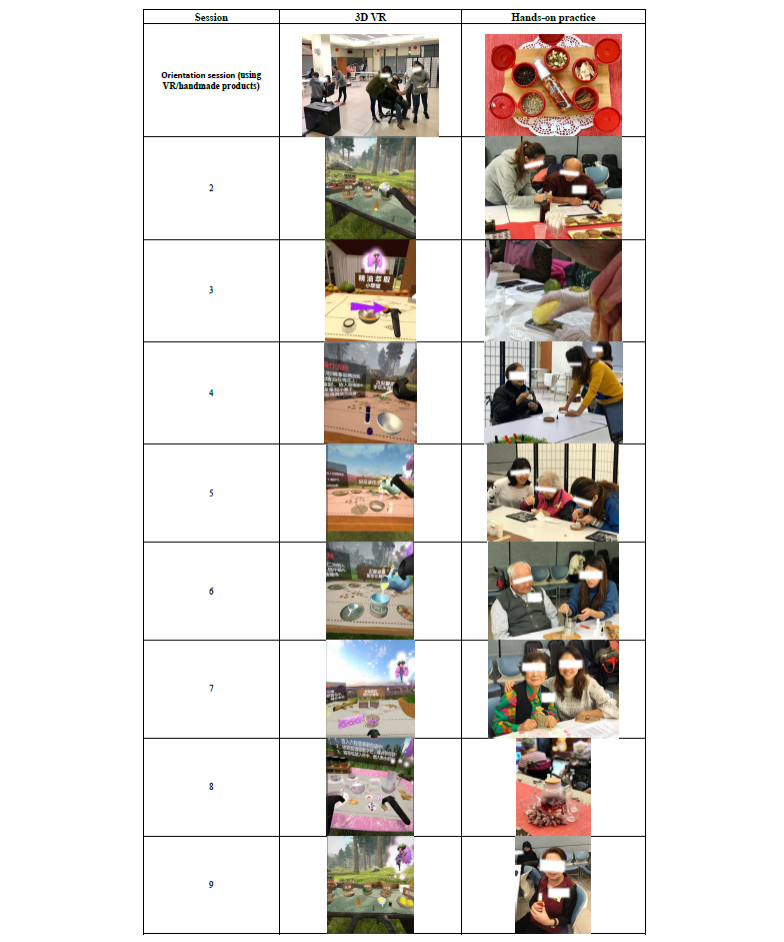
Examples of the 3D VR and hands-on aromatherapy program. 3D: 3-dimensional. VR: virtual reality.

The intervention consisted of 2-hour weekly sessions over 9 weeks. The first week involved ice-breaking activities, during which the participants were taught to wear 3D VR helmets and operate VR handles with familiar VR scenes so that the participants could practice multiple times to avoid dizziness. The contents of the program are shown in Figure 2.

The research team developed the combination program, involving aromatherapy, long-term care, elderly health promotion, and health education professionals. The characteristics of the 3D VR and hands-on aromatherapy program were appropriate for the psychological status of the elderly participants. The final versions of each session (see Multimedia Appendix 1) were prepared after multiple careful revisions of the program. Each session was delivered over 2 hours once a week, and in each session, the participants received hands-on guidance for preparing an aromatherapy product that they could use for the next 7 days. Conducted by 2 trained aromatherapy professionals with certifications from both the UK International Federation of Aromatherapists and the US National Association of Holistic Aromatherapy, with graduate students working as facilitators, the intervention was designed to promote happiness, stress relief, sleep quality, meditation experience, and life satisfaction. The prior findings in the “Introduction” section provided the evidence that helped us determine the program components and select the responsive psychological outcomes in the study.

The participants in the experimental group were divided into multiple groups with a facilitator to enhance individual engagement and solve the problems associated with operating the 3D VR device. A staff member of the nursing home asked participants to record a 7-day log of daily usage of the hands-on aromatherapy product, seen in Figure 2. This log was aimed at increasing the intervention intensity of the program to secure the expected effects. The staff checked participants’ logs weekly during the intervention period. All the participants successfully finished the 8-week logs with the support of the assisting staff. The participants were paired with the same group members and facilitator throughout the intervention in order to build rapport. Facilitators received a 2-day training workshop (16 hours total) to acquire knowledge of 3D VR and aromatherapy skills to assist in the activities performed by the participants.

### Measures

Sociodemographic variables assessed at baseline included age, gender, and educational level. The psychological outcome variables are presented below.

#### Happiness

The Oxford Happiness Inventory was used to measure happiness. It consists of 29 items and assesses the following 7 concepts: positive cognition, social commitment, positive affect, sense of control, physical fitness, satisfaction with self, and mental alertness [25]. Each item is scored on a Likert-type scale ranging from 1 to 4, with higher scores indicating a higher level of happiness. The Cronbach α coefficient was .92 during its development [26] and .90 in this study.

#### Perceived Stress

The Perceived Stress Scale is a self-reported scale that measures the degree of stress experienced by an individual over the last month. In comparison with life events, it has shown good predictive validity for various health outcomes [27]. The scale consists of 14 items [27], 7 positive and 7 negative, and was translated into Chinese. Each item is scored on a Likert-type scale that ranges from 0 (never) to 4 (very often). The scale has been shown to have good internal reliability, with a Cronbach α coefficient of .84 during its development [27] and .85 in this study.

#### Sleep Quality

The Pittsburgh Sleep Quality Index [28] was used to measure sleep quality. It is an effective instrument used to measure the quality and patterns of sleep in older adults. It differentiates poor from good sleep by measuring 7 domains over the last month: subjective sleep quality, sleep latency, sleep duration, habitual sleep efficiency, sleep disturbances, use of sleep medication, and daytime dysfunction. The scoring plan was followed according to the description provided in a previous study [28].

#### Meditation Experience

A shortened version of the 10-item Experiences During Meditation (EOM-DM) scale [25] was used to measure the experience of meditation. The original EOM-DM contains the following 5 subscales: cognitive effects, emotional effects, mystical experiences, relaxation, and physical discomfort. The first 2 subscales were appropriate for our study and were thus selected to measure the meditation experience. Each item was scored on a Likert-type scale from 1 to 5, with higher scores indicating a higher level of experience during meditation. The Cronbach α coefficients for the 2 subscales were .83 and .87 during its development [25] and .90 and .89 in this study.

#### Life Satisfaction

The life satisfaction scale for older adults developed by the Health Promotion Administration of the Ministry of Health and Welfare [29] was used to measure participants’ life satisfaction. This scale is a short-form version containing 10 items from the 20-item Life Satisfaction Index A [30]. Items are scored as 0 (disagree) and 1 (agree). The total raw score ranges from 0 to 10, with higher scores indicating a higher level of life satisfaction. The reliability of the Kuder-Richardson Formula 20 was 0.72.

### Data Analyses

Descriptive analyses were conducted for demographic and outcome variables. A 2-tailed *t* test and a chi-square test were used to compare differences in age, education level, and gender between the experimental and control groups. Because the use of a fragmented univariate test may lead to an inflated overall type 1 error, Hotelling *T*^2^ test (multivariate 2-group test) was performed for group comparisons of the 5 outcome measures at baseline [31]. A generalized estimating equation (GEE) was used to investigate the effect of time point, group, and their interaction on the outcome variables; GEEs enable an understanding of the patterns of change and their effects at both the individual and group levels by estimating the average response of the population (the population average effect), rather than regression parameters that would enable prediction of the effect of changing one or more covariates on a given individual [32]. Statistical analyses were conducted using SPSS (version 20.0; IBM Corp).

After the first-round analysis, we found that all outcome variables showed significant improvements (*P*<.001). This indicated that we could conduct a further analysis for the smaller sample of participants aged 80 and older (very old adults) in order to explore the intervention effects for this group. This further analysis is meaningful because a growing number of residents in nursing homes are in this age group, and these results are important to secure information on the implementation of the combined program for this population.

## Results

### Sociodemographic Data

The participants’ average age was 83.03 (SD 7.6) years and 81.92 (SD 9.0) years in the experimental and control group, respectively. There were no statistically significant differences in participants’ education levels and gender distribution between the experimental and control groups. Hotelling *T*^2^ results revealed that the 5 outcome measures at baseline were not significantly different between the groups (*T*^2^=10.95; *F*_5,40_=2.00; *P*=.10). Since the overall result was not statistically significant, we did not analyze each outcome variable separately.

### Improvements in Outcome Variables

**Figure 3:**
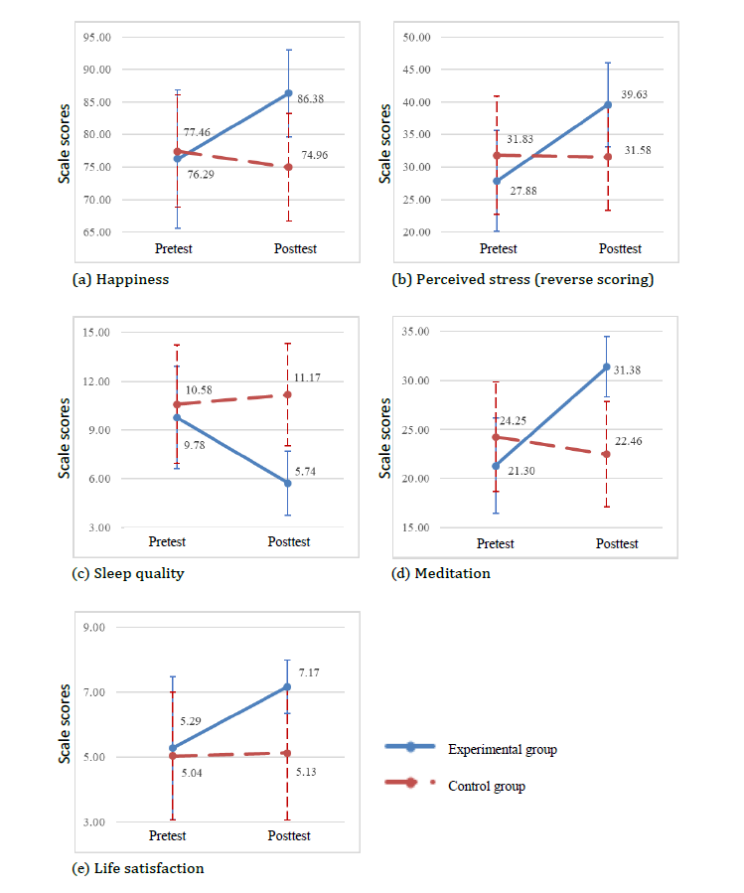
Changes in happiness, perceived stress, sleep quality, meditation, and life satisfaction between experimental and control groups.

Group differences in the patterns of change over time are shown in Figure 3. The results of GEE analyses indicated that the experimental group showed significant postintervention improvements in comparison with the control group in terms of the scores for happiness, perceived stress, sleep quality, meditation experience, and life satisfaction, as seen in Table 1. There was a significant group time interaction for the 5 outcome measures. The experimental group showed an improvement in the scores for happiness (GEE coefficient=12.58; *P*<.001), perceived stress (GEE coefficient=12.00; *P*<.001), sleep quality (GEE coefficient=–4.72; *P*<.001), meditation experience (GEE coefficient=11.92; *P*<.001), and life satisfaction (GEE coefficient=1.79; *P*<.001).

The majority of participants were adults aged 80 years and older (n=20 and n=15 in the experimental and control group, respectively), and the GEE analysis yielded a significant group time interaction for the 5 outcome variables. The experimental group showed an improvement in the scores for happiness (GEE coefficient=12.43; *P*<.001), perceived stress (GEE coefficient=13.00; *P*<.001), sleep quality (GEE coefficient=–4.46; *P*<.001), meditation experience (GEE coefficient=12.15; *P*<.001), and life satisfaction (GEE coefficient=1.55; *P*<.001).

**Table 1:** Results of generalized estimating equation analyses.

		GEE^a^ coefficient	SE	95% Wald CI			Wald chi-square (*df*)	*P* value
				Lower		Upper		
**Happiness**								
	Group (experimental group)^b^	–1.17	2.74	–6.53	4.20		0.2 (1)	.67
	Time (posttest)^c^	–2.50	0.79	–4.05	–0.95		9.9 (1)	.002
	Group (experimental group) × time (posttest)^d^	12.58	1.84	8.98	16.19		46.8 (1)	<.001
**Perceived stress (reverse scoring)**								
	Group (experimental group)^b^	–3.96	2.38	–8.63	0.72		2.8 (1)	.10
	Time (posttest)^c^	–0.25	0.73	–1.68	1.18		0.1 (1)	.73
	Group (experimental group) × time (posttest)^d^	12.00	1.72	8.64		15.36	48.9 (1)	<.001
**Sleep** **quality**								
	Group (experimental group)^b^	–0.72	0.97	–2.63		1.18	.6 (1)	.46
	Time (posttest)^c^	0.64	0.39	–0.12		1.40	2.7 (1)	.10
	Group (experimental group) × time (posttest)^d^	–4.72	0.65	–5.99		–3.45	53.2 (1)	<.001
**Meditation**								
	Group (experimental group)^b^	–3.01	1.50	–5.94		–0.07	4.0 (1)	.04
	Time (posttest)^c^	–1.79	0.59	–2.95		–0.63	9.2 (1)	.002
	Group (experimental group) × time (posttest)^d^	11.92	1.33	9.32		14.52	80.7 (1)	<.001
**Life satisfaction**								
	Group (experimental group)^b^	0.25	0.59	–0.90		1.40	0.2 (1)	.67
	Time (posttest)^c^	0.08	0.17	–0.24		0.41	0.3 (1)	.62
	Group (experimental group) × time (posttest)^d^	1.79	0.42	0.97		2.61	18.4 (1)	<.001

^a^GEE: generalized estimating equation.

^b^Reference group (group): control group.

^c^Reference group (time): pretest.

^d^Reference group (group time): control group pretest.

## Discussion

### Principal Findings

To our best knowledge, this is the first interventional study to use 3D VR and hands-on aromatherapy in a combined program and verify its effects on the psychological health of institutionalized older adults through an appropriate research method. A unique feature of this study is that the participating older adults engaged in hands-on preparation of aromatherapy products that they were then encouraged to use daily over the subsequent week. In addition, a research team member tracked the participants’ 7-day use of each aromatherapy product over 8 weeks. We believe that these strategies successfully increased the intervention intensity compared with previous studies, which often lacked a supporting strategy and tracking design for the daily use of hands-on aromatherapy products [33]. These results hold promise for the promotion of this combined aromatherapy program in nursing homes. In addition, the subgroup analyses targeting very old adults also demonstrated similar effects, which suggests that combined aromatherapy programs could be successfully adopted by decision-makers in nursing homes, given the recent sharp increase in the percentage of very old adults in these facilities. The number of very old adults is projected to increase more than threefold from 137 million to 425 million between 2017 and 2050 [2].

We used the outcome variable of happiness instead of depression, which was used in another study [34]. Therefore, we selected the Oxford Happiness Inventory, which is derived from the Beck Depression Inventory but contains more concepts. Congruent with previous studies, the current findings support the positive effect of short-term interventions on happiness among older adults. Tang and Tse [12] also found short-term improvements with hands-on aromatherapy for patients with depression. However, our posttest scores indicated that the intervention effect was observed after a 9-week intervention, which is different from a previous study [35] that only reported the short-term effect of aromatherapy for older adults with depression.

Moreover, we found an alleviating effect of aromatherapy on perceived stress, which is in line with a previous study [12] in which participants verbally reported stress relief after the first week of the 2-week aromatherapy intervention for stress management. The intervention effect observed in the posttest scores in our study indicated that the use of aromatherapy to decrease perceived stress in older adults is a promising approach. Aromatherapy could successfully reduce the levels of stress hormones and stimulate the production of β-endorphins [36]. For example, a previous study indicated that inhaling lavender and rosemary essential oils can increase free radical–scavenging ability and reduce cortisol secretion, relaxing muscles, relieving stress, and producing calmness [37].

Regarding sleep, a meta-analysis of 12 studies showed that the use of aromatherapy was effective in improving sleep quality [22], suggesting that readily available aromatherapy treatments appear to be effective in promoting sleep. Another systematic review and meta-analysis [13] including 31 trials with a randomized controlled design also indicated a significant effect of aromatherapy on sleep quality. In line with this, our participants reported a significant improvement in sleep quality (*P*<.001) in terms of posttest scores.

Smith and Kyle [38] indicated that the effects of aromatherapy are mediated by the stimulation of the limbic system by the chemical components or molecules of essential oils that are detected by the olfactory system after inhalation, which activates the hypothalamus and pituitary gland. Olfactory nerves then send signals to the limbic system to trigger memories and emotional responses and thus relieve mental stress.

However, in a systematic review and meta-analysis [13], 11 of 31 trials did not demonstrate any significant effects on sleep quality associated with the use of aromatherapy delivery modes such as inhalation, massage, and oral ingestion; the use of a blend of essential oils or a single essential oil; or the delivery with a mixed method (eg, acupressure massage). Thus, sleep quality interventions using aromatherapy should be administered on the basis of specific guidelines to ensure an efficient use of aromatherapy. We also propose that guidelines should be developed via a systematic approach by conducting appropriate research.

Regarding meditation, a previous study examining 20 adults’ meditation processes demonstrated electroencephalogram changes with lavender inhalation, which presented as an increase in fast theta and slow alpha activities in the frontal area during meditation [39]. Redstone [14] reported that meditation and aromatherapy caused a patient to say, “This is the first time I can sit for more than 5 minutes.” In line with the findings of previous research, our findings supported the significant effect of the combination of 3D VR and hands-on aromatherapy on the meditation experience. The effects of the combined program on the experience during meditation is an interesting topic that researchers can further explore in the future.

Aromatherapy has been found to be effective in improving psychological symptoms as well as overall quality of life, especially among patients with cancer [40]. We focused on a different study population, namely residents of nursing homes, which will have a wide application in the future. However, some previous studies have also indicated that aromatherapy did not improve life satisfaction [41] or quality of life [42] in the experimental group compared with the control group. These discrepancies might result from differences in the essential oils and targeted psychological variables in the studies. For example, Soden et al [42] used lavender essential oil and an inert carrier oil in their aromatherapy group, and their results were unable to demonstrate any significant long-term benefits of aromatherapy or massage in terms of improving quality of life.

A Cochrane systematic review [43] indicated that aromatherapy is commonly delivered with massage, which serves as complementary therapy that can reduce symptoms and improve the quality of life of patients with cancer. However, although the review found that there was some indication of benefit in the aromatherapy‐massage group, this benefit might not be clinically significant due to the small sample size of the studies. The contribution of our study is that it delivered a combination of 3D VR and hands-on aromatherapy to residents of nursing homes, which successfully enhanced their psychological health. Our findings are promising and could encourage researchers and practitioners to provide interventions combining technology and aromatherapy. Our findings suggest that aromatherapy confers benefits for psychological health. However, the methodologies employed in previous studies were heterogeneous, lacked replicable assessments and long-term follow-up, and employed small sample sizes. Replication, longer follow-up periods, and larger trials are critical in future research to accrue the necessary evidence for the research and development of aromatherapy.

### Limitations

First, because the program integrated 3D VR and hands-on aromatherapy, the contribution of either approach cannot be easily isolated using the current study design, as they are linked to each other. To validate the effectiveness of the 2 approaches separately, additional studies should be conducted using controlled trials with enough power. Second, we cannot comment on the longer-term effectiveness (ie, 12 months postintervention) of our program. Additional follow-up is needed to determine how the described intervention program affects older adults’ psychological health beyond 12 months after completion of the intervention. Third, the intervention might not apply to frail older adults, but it indicated that if more support is provided, such as the involvement of family members and caregivers, frail older adults could also participate in the program.

### Conclusion

The results are important for supporting similar future programs for institutionalized older adults. Our program adopted an innovative approach to improve psychological health among institutionalized older adults. A combination of 3D VR and hands-on aromatherapy activities provides more learning opportunities compared with other aromatherapy interventions. In addition, tracking the participants’ use of each aromatherapy product for 7-day periods over 8 weeks was a successful approach, as it extended the connection with participants outside the classroom and contributed to the significant improvements in psychological health among participants. In the future, the effectiveness of this approach may be scientifically verified by comparing 2 groups: those who receive only the aromatherapy program and those who receive the aromatherapy program and a monitoring approach such as usage logs.

To ensure early interventions for institutionalized older adults who experience psychological distress and to prevent the development of multiple psychological disorders, researchers and staff in nursing homes should target the older adults who are still in a state of psychological subhealth. Providing one-on-one interventions in nursing homes may not always be feasible or affordable. To reduce the burden of instruction, learning contents could be delivered through a 3D VR program, which can provide a safe and supportive learning environment and empower researchers and practitioners to play a vital role in solving problems, leading discussions, facilitating older adults’ learning, and providing feedback. During the intervention period, 2 staff members at the experimental nursing home were interested in participating in the delivery of the intervention and wished to act as facilitators to encourage participants to engage in the interventional activities. This indicates that our program can easily be implemented in nursing homes. Since the implementation is not complicated, nursing home staff receiving short-term training can deliver the program successfully to promote residents’ psychological health.

## Appendix

Multimedia Appendix 1 Combination of 3D VR and hands-on aromatherapy: Program components.

Multimedia Appendix 2 CONSORT-eHEALTH checklist (V 1.6.1).

## Abbreviations

3D: 3-dimensional
CAM: complementary and alternative medicine
EOM DM: Experiences During Meditation
GEE: generalized estimating equation
VR: virtual reality
