# Supplementary material for: Combination of 3-Dimensional Virtual Reality and Hands-On Aromatherapy in Improving Institutionalized Older Adults’ Psychological Health: Quasi-Experimental Study
Source: J Med Internet Res. 2020 Jul 23;22(7):e17096. doi: 10.2196/17096 (PMC7428146; doi:10.2196/17096)
Supplement: Multimedia Appendix 1 [file jmir_v22i7e17096_app1.docx]

Multimedia Appendix 1. Combination of 3D VR and hands-on aromatherapy: Program components

| Session | Purpose | Activity | Used essential oil | |
| --- | --- | --- | --- | --- |
| 1 | Breaking the ice | - Warm-up, introduction of program, and instructions to complete the usage log and operate the 3D VR device. | None | None |
| 2 | Use fragrance to access past experience and regain happy memories. | 3D VR:  Experience-building   - Introduction to herbs and aroma exploration - Linkage of herbal observation, breath and inhalation, and touch with happy memories - Instructions on preparation and use of herbal infused oils   Hands-on practice:   - Experience smell of herbs and share experiences - Make herbal infused oils | Mandarin, black pepper, cinnamon, sweet fennel, jasmine, ginger | Spices and citrus smell like warm sunshine to wake up, happy hour experience, boost pleasure and joy |
| 3 | Enhance pleasure by citrus aroma and immersion in aroma | 3D VR:  Experience-building   - Introduce a variety of citrus fruits and recall past happy experiences through olfaction   Task challenge   - Picking and counting citrus fruits - Extraction of sweet orange essential oil   Hands-on Practice:   - Extraction of sweet orange essential oil - Making citrus sprays | Mandarin, sweet orange, grapefruit, bergamot, lemon, pomelo | Spices and citrus smell like warm sunshine to wake up, happy hour experience, boost pleasure and joy |
| 4 | Smelling wood essential oils to balance the autonomic nervous system and relieve stress by immersing in the forest scene | 3DVR:  Experience-building   - Immersion in the forest scene, and sharing experiences of breathing in the forest - Experience the feelings in the forest to relieve stress - Instructions on preparation and use of blends of essential oils   Hands-on Practice:   - Share experiences of breathing in the forest with each other - Making forest scent snoring inhalers | Sandalwood, chamaecyparis, silver, rosewood, black spruce, cedarwood virginian | Forest scene with flowers and vast soothing, fragrant wood and flowers to release physical and mental stress. |
| 5 | Garden flourishing scene provides visual sensations and flower aromas, boosting mental and physical pleasure and reducing stress | 3D VR:  Experience-building   - Garden parade, enjoy six kinds of flowers   Task challenge   - Looking for unopened buds to bloom - Flower painting   Hands-on practice:   - Smelling the essential oils of flowers and sharing experiences. - Making floral cream | Rose damask, neroli, osmanthus, clary sage, tuberose, white champak | Forest scene with flowers and vast soothing, fragrant wood and flowers to release physical and mental stress |
| 6 | Describe resin harvesting and efficacy application, and smell resin essential oils to promote calmness and sleepiness | 3D VR:  Experience-building   - Demonstration of resin ointment production and explanation of the production steps - Demonstration of ointment on skin with massage   Task challenge   - Resin picking   Hands-on Practice:   - Making resin oil plaster for use before going to bed - Self-massage teaching, and share comfort experiences | Frankincense, myrrh, benzoin, storax, balsam copaiba, elemi | Plant resin can stimulate parasympathetic nervous system and is combined with lavender odor to improve sleep quality |
| 7 | Lavender field stroll and experiencing harmony with five senses, with physical and mental comfort | 3D VR:  Experience-building   - Explanation of picking, rubbing, and smelling the lavender spike   Task challenge   - Lavender bouquet picking - Scented bag production and application. Experience a 15-minute physical and psychological comfort sleep   Hands-on practice   - Hand-sniffing, rubbing lavender spike, and share experiences - Making a personal bedside scented bag | Lavender | Plant resin can stimulate parasympathetic nervous system and is combined with lavender odor to improve sleep quality |
| 8 | Roaming in and collecting herbs from a herb farm, enjoying herbs’ fragrance and experiencing meditation | 3D VR:  Task challenge   - Herb picking - Make herb tea bags   Experience-building   - Smell the aroma of herb tea and experience meditation   Hands-on practice:   - Making herb tea bags - Experience the aroma and taste of herb tea. | Licorice, peppermint, verbena, chamomile roman, butterfly pea, stevia | Herb energy is diverse, use tea flavors and smells to calm anxiety, blending essential oil massage to achieve relaxation in meditation |
| 9 | Recognize the meditation-related plants, smell aroma of plants and practice meditation | 3D VR:  Experience-building   - Introduction to meditation-related plants - Experience the aroma of meditation-related plants and practice 15-min meditation   Experience-building   - Making meditation aroma ball bottle   Hands-on practice:   - Experience the aroma of meditation-related plants and share experiences - Making meditation aroma ball bottle | Sweet orange, elecampane, black spruce, frankincense, sandalwood, patchouli | Herb energy is diverse, use tea flavors and smells to calm anxiety, blending essential oil massage to achieve relaxation in meditation |
